# Disgust versus Lust: Exploring the Interactions of Disgust and Fear with Sexual Arousal in Women

**DOI:** 10.1371/journal.pone.0118151

**Published:** 2015-06-24

**Authors:** Diana S. Fleischman, Lisa Dawn Hamilton, Daniel M. T. Fessler, Cindy M. Meston

**Affiliations:** 1 Department of Psychology, University of Portsmouth, Portsmouth, Hampshire, United Kingdom; 2 Department of Psychology, Mt. Allison University, Sackville, New Brunswick, Canada; 3 Department of Anthropology and Center for Behavior, Evolution, and Culture, University of California Los Angeles, Los Angeles, California, United States of America; 4 Department of Psychology, The University of Texas at Austin, Austin, Texas, United States of America; Catholic University of Sacred Heart of Rome, ITALY

## Abstract

Sexual arousal is a motivational state that moves humans toward situations that inherently pose a risk of disease transmission. Disgust is an emotion that adaptively moves humans away from such situations. Incongruent is the fact that sexual activity is elementary to human fitness yet involves strong disgust elicitors. Using an experimental paradigm, we investigated how these two states interact. Women (final *N*=76) were assigned to one of four conditions: rate disgust stimuli then watch a pornographic clip; watch a pornographic clip then rate disgust stimuli; rate fear stimuli then watch a pornographic clip; or watch a pornographic clip then rate fear stimuli. Women’s genital sexual arousal was measured with vaginal photoplethysmography and their disgust and fear reactions were measured via self-report. We did not find that baseline disgust propensity predicted sexual arousal in women who were exposed to neutral stimuli before erotic content. In the Erotic-before-Disgust condition we did not find that sexual arousal straightforwardly predicted decreased image disgust ratings. However, we did find some evidence that sexual arousal increased self-reported disgust in women with high trait disgust and sexual arousal decreased self-reported disgust in women with low trait disgust. Women who were exposed to disgusting images before erotic content showed significantly less sexual arousal than women in the control condition or women exposed to fear-inducing images before erotic content. In the Disgust-before-Erotic condition the degree of self-reported disgust was negatively correlated with genital sexual arousal. Hence, in the conflict between the ultimate goals of reproduction and disease avoidance, cues of the presence of pathogens significantly reduce the motivation to engage in mating behaviors that, by their nature, entail a risk of pathogen transmission.

## Introduction

Consonant with the adaptive significance of avoiding disease, humans generally shun situations that present cues of potential disease transmission. However, in some contexts such avoidance is incompatible with achieving other adaptive goals. Sexual behavior is prototypical in this regard, as mating success is an elementary determinant of fitness, yet sex inherently involves extensive exposure to stimuli that index disease risk. Given that emotions and related motivational states can be understood as evolved mechanisms, each of which serves a specific adaptive goal [1,2] it is therefore possible that disgust and sexual arousal may constitute motivational engines that work against [3–8] one another.

There is general agreement that a core adaptive function of disgust is to reduce the risk of contracting disease by distancing the actor from cues of the presence of pathogens. Body odors and secretions normally encountered in sexual contexts are some of the strongest disgust elicitors [9]. Moreover, sexual behavior entails not only increased contact with cues of disease, but also increased vulnerability to disease. In addition to the direct exchange of bodily fluids, the exposure of delicate mucous membranes—along with possible attendant abrasion—presents a ready entry opportunity for microorganisms, while increased respiration in conjunction with close proximity enhances the risk posed by airborne pathogens. By virtue of the fact that disgust motivates avoidance, there is thus an inherent conflict between intrinsic features of sexual behavior and core, or pathogen-avoidance, disgust [10]. In this study we investigate how sexual arousal and disgust influence one another in women, employing both self-report instruments and a physiological measure of sexual arousal.

Pathogen avoidance or core disgust has been found by disgust measures to be distinct from what has been called the sexual domain of disgust [11]. The sexual domain of disgust can be understood as an adaptation serving the ultimate function of reducing suboptimal mating behavior [8]. Though distinct, sexual disgust (e.g. the disgust experienced when considering incest), [8,12] often overlaps with pathogen disgust (e.g. when cues of infection reveal low genetic quality) [10]. However, in this study we examine the conflict between pathogen-avoidance disgust and sexual arousal, rather than examining the conflict between uniquely sexual disgust and sexual arousal.

### Functionality in Women’s Heightened Disgust Propensity

Disgust propensity is defined as “a general tendency to respond with the emotion of disgust to any given situation” [13]. One of the most consistent findings in the disgust literature is that women tend to have a heightened disgust propensity relative to men, especially in the sexual domain [3,14–17]. Women also are more likely to implicitly associate sexual images with disgust than men [16]. While a number of factors likely contribute to this sex difference, it may have notable adaptive significance in regard to the disease risks posed by sexual behavior. Women have a greater area of mucous membranes and experience more tissue trauma during coitus, making them more susceptible to sexually transmitted infections than heterosexual men [18]. With regard to almost all sexually transmitted infections, women who engage in heterosexual intercourse also suffer a greater disease burden than heterosexual men. Progesterone-driven cyclic reductions in female inflammatory responses, which reach their peak during the luteal phase of the menstrual cycle, increase female vulnerability to infection (reviewed in [19,20]). Additionally, human female anatomy makes it possible for pathogenic bacteria to travel through the vagina into the peritoneal cavity, causing pelvic inflammatory disease (PID); of women with a single episode of PID, 8% are rendered infertile [18] representing one of the most severe adaptive costs.

### How Sexual Arousal Influences Disgust Reactions

To date, four studies, two with male samples [21,22], one with a female sample [23] and one with both male and female participants [24] have specifically investigated whether people respond differently to disgust elicitors when sexually aroused. Ariely and Lowenstein [21] found that, compared to men who viewed pictures of clothed women, those who viewed pictures of naked women were significantly more likely to endorse engaging in potentially disgusting sexual situations, such as watching a woman urinating, having sexual contact with an animal, or having anal sex. Stevenson, Case and Oaten [22] used disgust stimuli from each of three modalities (aural, visual and tactile), with one sex-related stimulus and one non-sex-related stimulus presented in each modality (e.g., for the tactile modality: feeling lubricated condoms versus feeling cold ham and pea soup). Consistent with Ariely and Loewenstein’s [21] findings, compared to men in control conditions, men who had been exposed to erotic content (photographs of nude women) showed a greater discrepancy between disgust reactions to sexual and nonsexual stimuli. Specifically, sexual arousal reduced disgust responses for sex-related stimuli, but had no effect on disgust reactions to non-sexual stimuli [22].

Another study did not find that sexually aroused men reported a decrease in disgust. Lee et al. [24] conducted an online study examining how men and women responded to the Three Domains of Disgust Scale (TDD; [11]) in a sexually aroused state, a physiologically aroused state or a control (no arousal) state. Participants achieved sexual arousal in their preferred way at home. The sexual arousal condition did not reduce the disgust of male participants in any of the three domains of disgust (pathogen disgust, sexual disgust or moral disgust) [24]. This finding is at odds with previous studies [21,22] perhaps indicating that young men did not comply with instructions to just achieve sexual arousal and not orgasm before returning to the survey.

In light of the above findings in men, and given the adaptive considerations discussed earlier, it is plausible to expect sexual arousal to influence women’s reactions to disgust-eliciting stimuli. Borg & de Jong looked at how women respond to engaging in disgusting tasks when in one of three mood-induction groups: positive arousal, negative arousal, and sexual arousal [23]. Women watched 5 minutes of video intended to elicit one of these three states and then conducted 2 of 16 disgusting tasks (rather than actually doing the task, participants could also choose to imagine engaging in it). This procedure (5 minutes of video and 2 new disgusting tasks) was repeated until each participant had been exposed to all 16 disgusting tasks. Compared to women in both the positive arousal and the control conditions, women in the sexual arousal condition reported less disgust at engaging in (or imagining engaging in) the sexual disgusting tasks (e.g., lubricating a vibrator, handling a pair of stained underwear), thus replicating in women Stevenson et al.'s [22] findings in men. Additionally, Borg and de Jong [23] found a nearly significant difference in disgust reactivity for the nonsexual disgusting tasks (e.g., inserting a pin into a cow eyeball), such that women in the sexual arousal condition reported less disgust than those in the positive arousal condition. This study also found that those in the sexual arousal condition completed the most disgusting tasks compared to the neutral or positive arousal condition regardless of whether these tasks were sexual in nature. This study provides evidence that both pathogen disgust and sexual disgust may be attenuated by sexual arousal.

Lee et al. [24] found the same effect as Borg & de Jong [23] with regards to sexual disgust but the *opposite* effect for pathogen disgust in their online study. Replicating Borg & de Jong, sexually aroused women reported being less disgusted at contexts in the sexual domain of the TDD scale (e.g. “Finding out someone you don’t like has sexual fantasies about you”) than women in the physiologically aroused and control conditions. However, sexually aroused women reported significantly *more* disgust at the pathogen disgust contexts in the TDD (e.g. “Stepping on dog poop”) than women in the physiologically aroused condition and marginally more pathogen disgust than women in the control condition.

### How Disgust Influences Sexual Arousal

There have been studies of how trait disgust influences sexual arousal and functioning (e.g. [16,25]) but no studies have really examined how *eliciting disgust* directly influences sexual arousal. Koukounas and McCabe [26] found that women reported more disgust than men at erotic videos and that these disgust ratings were associated with significantly lower self-reported sexual arousal. However, this result does not distinguish between core disgust and moral disapproval expressed as disgust, an important confound given that disapproval of pornography in general, or of gender roles depicted therein, could also create this effect. Malamuth and Check [27] asked male participants to read stories of sexual encounters in which the degree of female arousal and consent depicted were manipulated, finding that vignettes in which the woman was described as disgusted were less subjectively arousing than those in which she was described as sexually aroused. However, this study addresses observed disgust in another party rather than own disgust, while the absence of a neutral condition makes it impossible to determine whether observed disgust exercises a unique effect on participant arousal. Vonderheide and Mosher [28] found that the more disgust women reported when imagining inserting a contraceptive diaphragm, the less sexual arousal they reported while imagining a subsequent sexual interaction. However, the same study suggests that both the elicited disgust and the associated reduced arousal likely reflect underlying negative attitudes toward sexuality. Ethnographic accounts describe an inverse relationship between disgust and sexual arousal among the Mangaians of Polynesia [29] and the Bengkulu of Sumatra [30], but it is impossible to distinguish personal experience from folk models in such reports. Disgust has also been found to have an effect on hypothetical decision making in a sexual context: participants exposed to the smell of feces reported being more likely to use condoms [31]. Murray, Jones & Schaller [32] found a correlation between self-reported vulnerability to disease, lower number of desired future sexual partners and reduced future promiscuity. This correlation was stronger in a group of women who had been exposed to disease cues. However, both results reveal changes in sexual-decision making in the presence of disease cues rather than a decrease of sexual arousal per se. Hence, to date, there have been no direct tests of the effects of experimentally elicited disgust on subsequent sexual arousal.

Clinically, trait disgust has been shown to have implications for women’s sexual functioning. Women with vaginismus (a condition in which vaginal spasms make intercourse difficult or impossible) were found to have greater overall disgust propensity (as measured using the Disgust Scale (DS)–[14]) than women with dyspareunia (genital pain related to intercourse) and women without sexual complaints [33]. Surprisingly, this study showed no overall differences between groups on ratings from a Sexual Disgust Questionnaire (e.g., “To what extent are you willing to lie beneath bedclothes in a hotel that look unwashed, and below which previous guests may have had sexual intercourse?”) [33]. A follow-up study found that women with vaginismus and dyspareunia both showed greater implicit disgust associations to sexual stimuli, and that women with vaginismus showed greater facial muscle activation reflecting disgust when viewing an erotic film [25].

Researchers have also looked at the relationship between trait disgust and sexual functioning in nonclinical samples. van Overveld et al. [34] found that a) lower willingness to handle sexual items from a familiar source, and b) greater disgust at handling such items significantly predicted lower ratings on the subjective arousal and lubrication subscales of the Female Sexual Functioning Index. Grauvogl et al. [16] reanalyzed this data [34] finding that those women with greater disgust propensity across domains also reported experiencing more pain during intercourse and had more difficulty achieving orgasm whereas for men trait disgust was not associated with sexual functioning. In a further study, Grauvogl et al. [16] brought 19 men and 24 women into the laboratory. They looked at the association between genital sexual arousal (measured with psychophysiological instruments) and three disgust measures: implicit association between sexual stimuli and disgust, disgust propensity and the tendency to find the emotion of disgust unpleasant. They did not find any association between disgust measures and genital sexual arousal in women. Surprisingly, they found that higher disgust propensity and unpleasantness was associated with *greater genital arousal* in male participants. Thus, there does not seem to be good evidence that trait disgust reduces genital response to sexual stimuli.

### Fear and Sexual Arousal

Although the effects of elicited disgust on sexual arousal have not been directly examined, the effects of anxiety and stress have. A review of this literature informs predictions for the fear condition that we employ as a negatively-valenced control condition in the present study. “Stress” is fairly loosely defined in the literature and, consistent with this lack of precision, has been shown to have a variety of effects. Hoon, Wincze, and Hoon [35] found that a physiological marker of sexual arousal was elevated when an erotic film clip was presented after an anxiety-inducing clip (depicting dying victims in the aftermath of an automobile accident) compared to when the erotic clip was presented after a neutral clip. Palace and Gorzalka [36] replicated these findings with two pairs of films: a neutral film followed by an erotic film, and an anxiety-inducing film followed by an erotic film; women showed more vasocongestion (indicative of arousal) during the erotic film shown after the anxiety-inducing film. In studies where a stressor leads to increased physiological arousal, the facilitatory effect is likely due to increases in sympathetic nervous system activity, which has been shown to enhance subsequent arousal [37,38]. However, self-reported sexual arousal appears not to follow the same pattern. Palace and Gorzalka [36] found that, rather than reporting more sexual arousal following exposure to anxiety-inducing stimuli, women in this condition reported less arousal, while other studies show activation of the sympathetic nervous system does not change self-reported arousal [37,38]. The former pattern is potentially explicable in terms of subjective misattribution [39] a possibility that, as we discuss below, has important methodological implications. In contrast to these findings, stress that entails the threat of harm has been shown to decrease sexual arousal. For example, women who were told that they had a 60% chance of receiving a painful electric shock showed lower genital arousal than did women in a control condition [40]. Frustration, such as a cognitively frustrating task with a researcher present, also lowers sexual arousal [41]. Importantly, all of the myriad fear and anxiety induction methods employed by previous investigators differ substantially from the stimuli that we employ. In order to invoke a negatively-valenced emotional state that is functionally divergent from disgust, we specifically selected images of situations that can plausibly be presumed to have potential adaptive significance for a human observer, yet do not include cues of the risk of disease transmission (e.g., predators, precipitous heights, natural disasters, weapons).

Can disgust also be construed as a stressor? Disgust appears to involve coactivation of the sympathetic and parasympathetic nervous systems, rather than exclusive activation of the former [42]. Hence, in contrast to the predictions of the adaptationist account presented earlier, at a proximate level, the potential impact of disgust on sexual arousal is unclear, as such influence may depend on the ratio of SNS to PNS activation.

### The Current Study

Here we present the first study to directly measure the impact of disgust on sexual arousal and vice versa using both self-report of sexual arousal and physiological indices of sexual arousal. Previous studies have generally used a measure of subjective sexual arousal. Self-report measures of women’s sexual arousal often show low correlation with genital measures of sexual arousal (i.e., vaginal pulse amplitude, the measure used in this study) [43] or even a negative correlation [16]. Self-reported sexual arousal may represent higher-order conscious appraisal of sexual cues, while genital measures may be the product of more immediate and unconscious processing [44,45]. Given that self-reported sexual arousal may thus reflect a greater influence of the cultural meaning of particular sexual cues, while genital arousal may reflect more elementary features of biological responses to such stimuli, it is possible that genital arousal will more clearly reveal the traces of evolved mechanisms designed to weigh the competing adaptive goals of reproduction and disease avoidance. By measuring both genital and self-reported sexual arousal, the present study allows for a more comprehensive view of the relationship between disgust and sexual arousal in women.

### Predictions and Rationales

Because, over evolutionary time, the experience of disgust has been a reliable indicator that a propitious mating is unlikely, we predict that disgust will decrease sexual arousal, and hence that participants in whom disgust is elicited prior to sexual arousal will show the lowest sexual arousal. Disgust propensity appears to be calibrated to current immune vulnerability [46] and thus with increased probability of contagion during sexual contact. We therefore predict that baseline disgust propensity will predict lower overall sexual arousal in our participants. Because physiological measures of sexual arousal represent a more basic and unconscious processing of erotic cues, we predict that this measure will be more susceptible to the effects of disgust induction than will subjective indicators of sexual arousal.

With regard to the effects of sexual arousal on core disgust in women Borg and de Jong [23] found that sexual arousal significantly decreased core disgust reactions in comparison to a neutral condition. Lee et al. [24] found that sexual arousal increased pathogen disgust in women relative to a physiologically aroused control condition. However, both groups of investigators measured sexual arousal through self-report, leaving open the question of the extent to which these patterns reflect symbolic meanings versus more physiologically-driven processes. Here, we pit two predictions against one another. On the one hand, sexual arousal could *decrease* pathogen-avoidance disgust because the ultimate adaptive function that sexual arousal serves—achieving reproductive success through mating—can only be achieved through intimate contact with cues of pathogen presence. On the other hand, because sexual arousal (especially when measured in terms of vasodilation, explained below) potentially corresponds with increased risk of contagion, sexual arousal could *increase* disgust reactivity.

Finally, because previous work, characterized by heterogeneous methods and divergent stimuli, documents mixed effects of stress and anxiety on subsequent sexual arousal, we have no discrete predictions regarding the effects of fear induction in this regard. Rather, we treat our fear condition as a negatively-valenced control for our disgust condition, with the understanding that the former is itself exploratory in nature.

## Data Archiving

Data for all studies described in this paper are archived at the Dryad Digital Repository.

## Method

### Ethics Statement

The research was approved by both the ethics boards at the University of Texas at Austin and that at Mount Allison University. During screening, informed verbal consent was obtained from participants. On the day of the experiment, informed written consent was obtained from all participants. Written consent forms were retained and locked in a secure cabinet.

### Screening Participants

Potential participants were screened by phone using the following eligibility criteria: a) female over age 18, b) heterosexual, and c) experiencing regular menstrual cycles. Exclusionary criteria were as follows: a) self-reported sexual aversion or distress related to sexual abuse, b) not having consistent menstrual cycles (e.g., pregnant, menopausal), c) breastfeeding within the last 3 months, d) any untreated endocrine disease, and e) taking medications that could interfere with the study, including DHEA, SSRIs or beta blockers. Women on hormonal contraception participated in the study. After the initial phone screening, participants were scheduled to come into the lab between days 5–10 of their menstrual cycle in order to standardize menstrual cycle effects.

### Participants

Participants were members of the undergraduate psychology subject pool at a North American university, receiving course credit in exchange for participation, or were compensated volunteers from North America, recruited via Craigslist.org in exchange for $15. Eighty-four women participated in the study. We excluded data from those participants for whom we were unable to obtain a valid vaginal pulse amplitude (VPA) reading (n = 6), one woman who was an outlier (discussed later), and one woman who suffered conjunctivitis at the time of participation, leaving 76 participants total. Participants ranged in age from 18–42 (mean = 23.55, SD = 5.44). The final sample was 55% White, 14.5% East Asian, 9.2% African American, and 10.5% Latina, 6.6% Mixed Latina and White; additionally, there were two South Asian participants, and one participant who did not indicate her ethnicity. The sample consisted of 48 women not using hormonal contraceptives, 27 women using hormonal contraceptives, and one woman who did not answer this question.

### Pre-Experiment Materials

#### Questionnaires and background materials

All questionnaires were completed before the experiment began. Individual differences in pathogen-avoidance disgust propensity were assessed using a six-question subset of the Three Domains of Disgust Scale (TDD; [11]). We used this truncated version of the TDD because we did not want to elicit strong disgust—especially sexual disgust—in participants before the experiment began. The six-question subset exhibited a reasonably good alpha in the present study α (76) = .72, akin to that (alpha α [1064] = .78) characterizing these items in the original Tybur et al. [15] study in which the TDD was developed [47]. Participants also filled out the Female Sexual Function Index [48], as well as questionnaires regarding their health, position in the menstrual cycle, and relationship status. Additional questionnaires, addressing other topics, were also administered but are not discussed here.

### Experimental Design

**Fig 1:**
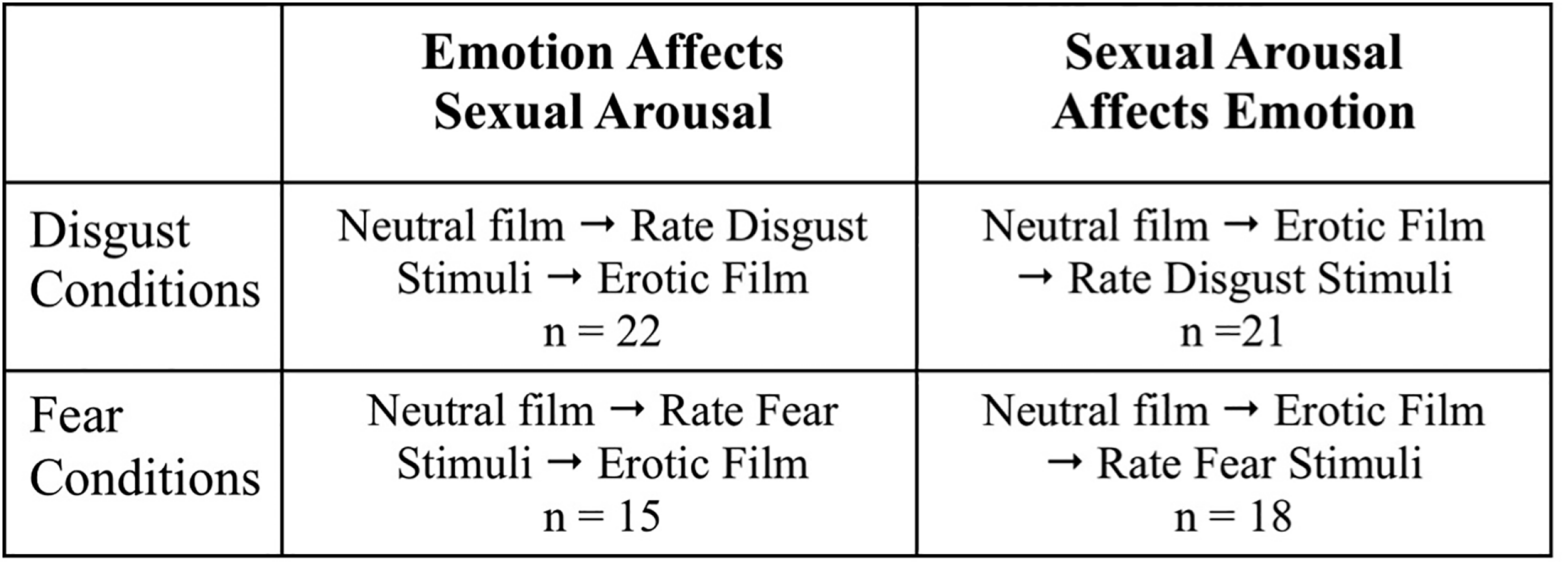
Four conditions were employed, as follows (clockwise from the upper left hand corner). **Disgust affects sexual arousal**: Women rated 18 disgust-inducing pictures and then watched an erotic film; **Sexual arousal affects disgust**: Women watched the erotic film first, then rated 18 disgust-inducing pictures; **Sexual arousal affects fear**: Women watched the erotic film and then rated 18 fear-inducing pictures; **Fear affects sexual arousal**: Women rated 18 fear-inducing pictures and then watched the erotic film. The two fear conditions control for the contributions of negative valence, an attribute shared by disgust and fear.

The study design is illustrated in Fig 1.

### Films

#### Neutral film

The neutral film was a 3-minute clip, including audio, from a documentary about the search for the naval ship PT-109; the clip features mostly footage of boats, along with some interviews.

#### Erotic film

The erotic film was an 8-minute clip drawn from the Female Sexual Psychophysiology Laboratory film library. All films in this library have been standardized in terms of the length of different types of sexual scenes (i.e., foreplay, oral sex, and vaginal intercourse). None of the films show sexual violence or fellatio. These films were selected from erotic films produced and directed by women, and are intended to be sexually appealing to women. Participants were each shown one such film. We chose to use films rather than still images because audiovisual depictions of intercourse have been shown to elicit greater genital arousal in women than still images of nude men and women [43].

### Disgust and Fear Induction

#### Creating the stimuli set

Stimuli intended to elicit fear and disgust were taken from internet searches, the images in the Curtis disgust inventory [3] and the International Affective Picture System [49]. Four raters (two men and two women) each rated 194 images with regard to how frightening, enjoyable, sexual, and disgusting each was on a scale from 1–9. We excluded images that were ranked as both highly disgusting and highly sexual because we wanted to ensure that the disgust stimuli would neither arouse participants nor specifically elicit sexual disgust. We then subtracted the fear rating from the disgust rating, yielding 18 images with the highest disgust rating independent of fear rating. We performed a similar assessment of fear images, subtracting disgust rating from fear ratings, and selecting those 18 images having the highest fear ratings independent of disgust ratings.

#### Description of fear and disgust inducing stimuli

The images in the disgust slideshow comprised 9 photographs of infected, diseased, or injured humans or human corpses, 3 photographs of feces, 5 pictures of dead and rotting animals or animal parts, and one photograph of a human vomiting. The images in the fear slideshow comprised 7 photographs of weapons or violent humans, 7 photographs of dangerous animals (e.g. wolves, sharks), and 4 photographs of hazardous environments (e.g. heights, fire, tornado). We chose to use still images because they offered a breadth of disgust induction and because, as far as we are aware, there is no evidence that films are better at inducing disgust than still images. Two participants did not fill out the image ratings at all, and two participants only filled out image ratings for two out of the four valence options. These participants are excluded only for those correlations in which their data was missing. The mean disgust rating of the disgust images for those 43 women in the two disgust conditions was 4.88 (1.50) out of a possible 9, and the mean fear rating of the fear images for those 32 women in the two fear conditions was 3.92 (1.52) out of a possible 9.

#### Image ratings

Picture Rating Sheet- Participants viewed each disgust- or fear-inducing image for 8 seconds, then had 8 seconds to record their evaluation of it on a pre-printed paper rating sheet, using 1–9 scales. They rated each image on how fearful, disgusting, sexual, and enjoyable it was.

### Measurement of Sexual Arousal During the Erotic Film

#### Apparatus for vaginal measures

Vaginal Photoplethysmograph. Genital arousal in response to all films was measured using a vaginal photoplethysmograph [50], a clear acrylic tampon-shaped device that contains an infrared light-emitting diode as a light source, and a photosensitive light detector. The measure of interest from the photoplethysmograph is the vaginal pulse amplitude (VPA), which is received through the A/C signal and band pass filtered at. 5 to 30 Hz. VPA was sampled 80 times per second. Results are measured in millivolts (mV). VPA data were acquired using the software program AcqKnowledge III, Version 3.7.3 (BIOPAC Systems, Inc., Santa Barbara, CA) and a Model MP100WS data acquisition unit (BIOPAC Systems, Inc., Santa Barbara, CA) for analog/digital conversion.

#### Self-report measures of arousal

*Self-report Sexual Arousal*. *Self-report* sexual arousal was continuously monitored throughout the duration of the erotic film using a device consisting of a computer mouse mounted on a track numbered from 0 to 7; the device was positioned on a small table a comfortable distance from the side of the participant's chair. The participant began with the device set at zero and moved the mouse up or down to indicate increasing or decreasing self-reported arousal during the erotic film presentation. The participant was instructed that the device was used to measure their psychological arousal, and that zero indicated no arousal, and 7 indicated intense sexual arousal. The levels of sexual arousal indicated and the amount of time spent at each level of arousal were recorded and analyzed using the Matlab software package (student version, release 12). The output for this “arousometer” is measured as a proportion of the maximum. We extracted three variables from these data: the mean level of self-report sexual arousal during the erotic film, the maximum level of self-report sexual arousal achieved during the erotic film, and the length of time it took participants to achieve their peak self-reported sexual arousal.

### Procedures

The study procedures were explained to the participant, including the proper insertion of the vaginal photoplethysmograph and the placement of electrode wires. After the participant read and signed the consent form, three electrodes were applied to her skin for electrocardiography (to aid in analysis of VPA), and the participant was left alone to insert the photoplethysmograph and fill out the surveys. All further communication with the participant took place via intercom. Participants viewed the experimental stimuli on a 42-inch plasma screen television mounted on the wall. All four experimental condition sequences were approximately 15 minutes long, and all began with a one minute display of the word “Relax” on a black screen, followed by three minutes of the neutral film, and then the remainder of the experimental protocol described above.

### Data Analysis

#### Preprocessing

*VPA Data*. VPA data were reduced by calculating the total change in amplitude for each heartbeat. This was done by finding the peak and nadir for each pulse wave and computing the difference between the two to obtain the amplitude for each pulse wave, using AcqKnowledge software. Artifacts in the data were identified visually by the researcher and removed manually. VPA is a relative measure with no true zero. Thus, in keeping with previous investigations (e.g. [51,52]) VPA was averaged across each condition and then a score for each participant for each condition was calculated as percent change during the experimental film relative to the neutral film that followed it. This variable will be referred throughout the paper as VPA percent change.

## Results

### Exclusions

One outlier was removed from the study: a participant in the Disgust-before-Erotic condition showed a percent change of 178.55. This was 3.97 standard deviations from the mean for the sample as a whole and 4.08 standard deviations from the mean within the Disgust-before-Erotic condition. Two other participants, both in the Fear-before-Erotic condition, showed percent change greater than 3 standard deviations from the mean for the sample as a whole. However, these percent changes were only 2.4 and 2.3 standard deviations from the mean within the Fear-before-Erotic condition, and hence data from these participants were not excluded.

### TDD Scale

Three participants did not complete the TDD subset. Scores on the TDD subset did not differ significantly across conditions both when all four conditions were examined separately *F*(3,68) = 2.36, *p* = .08 and when the two neutral conditions (Neutral-before-Erotic) were combined *F*(2,69) = 2.31, *p* = .11. The TDD scores for the two disgust conditions were nearly identical to one another (see Table 1). We found the TDD subscale of disgust propensity we used to be highly correlated with disgust image ratings *r*(39) = .44, *p* < .01, thus validating the measure. Because differences in values on the TDD subscales are so close to significant, results controlling for TDD are also reported where appropriate.

**Table 1:** Descriptive statistics for participants divided by condition.

	Disgust before Erotic	Erotic before Disgust	Fear before Erotic	Erotic before Fear
n	22	21	15	18
Age	24.32 (4.74)	22.57 (5.58)	23.20 (5.87)	24.06(5.96)
Percent change	10.87 (18.35)	37.09 (32.05)	35.54 (50.97)	25.47 (27.40)
n	22	21	15	16
Disgust Reactivity (image ratings)	4.78 (1.37)	4.99 (1.66)	1.90 (0.68)	1.28 (0.41)
n	22	21	15	17
Fear Rating	3.59 (2.04)	4.40 (2.43)	4.38 (1.11)	3.51 (1.74)
n	20	21	15	16
Disgust Propensity Score (TDD)	3.51 (1.32)	3.31 (1.14)	3.74 (1.16)	2.73 (.66)
n	19	20	11	13
Mean self-reported arousal	.43 (.11)	.44 (.15)	.44 (.16)	.45 (.17)
Time to peak self-reported arousal (in seconds)	384 (81)	316 (104)	353 (103)	327 (148)
Maximum self-reported arousal	.67 (.18)	.68 (.17)	.66 (.19)	.65 (.22)

TDD = Three domains of disgust scale. Participants in the two disgust conditions were only exposed to disgust inducing stimuli and participants in the latter two fear conditions were only exposed to fear eliciting images. Disgust ratings and fear ratings represent ratings for each condition’s image set.

### Correlation of Self-Reported Arousal and VPA Percent Change

Self-report sexual arousal was not recorded for 13 participants in the final sample. Nine participants forgot to use the arousometer or used it incorrectly, in three cases the equipment failed, and in one case it is unclear why results were not recorded. Among the 64 participants for whom such data were recorded, we assessed three different measures: average self-reported arousal across the entire erotic film clip, maximum self-reported sexual arousal, and elapsed time to peak self-reported sexual arousal. The descriptive statistics for the self-reported sexual arousal ratings are presented in Table 1. Similar to other studies, self-reported arousal did not correlate significantly with genital arousal [43]. Across the two control conditions (where neither fear inducing stimuli or disgust stimuli were presented before the arousing film clip) correlations between VPA percent change and self-reported arousal were positive: average self-reported arousal, *r*(31) = .15, *p* = .41, maximum self-reported sexual arousal, *r*(31) = .20, *p* = .26 and elapsed time to peak self-reported sexual arousal *r*(31) = .03, *p* = .89.

### Experiment-Specific Tests and Predictions

Below we detail specific predictions about the effects of emotion inductions, ratings, and sexual arousal as measured through VPA and presented as percent change. For those predictions made *a priori* we have used one-tailed tests. Given the vagaries of self-report sexual arousal (due to misattribution, etc.), we had no specific predictions regarding this variable, but nevertheless present these results alongside the physiological measures for exploratory purposes. We make the following important terminological distinction: *disgust propensity* refers to the results of the questionnaire measure administered before the study (six questions from the pathogen subset of the TDD); in contrast, *disgust ratings and disgust reactivity* refers to participants’ assessments of the extent to which the visual disgust stimuli were perceived as disgusting.

Prediction 1- If disgust inhibits sexual arousal, then participants in Condition 1 (Disgust-before-Erotic) will show the lowest physiological sexual arousal (measured as percent change) relative to participants in other conditions.

**Fig 2:**
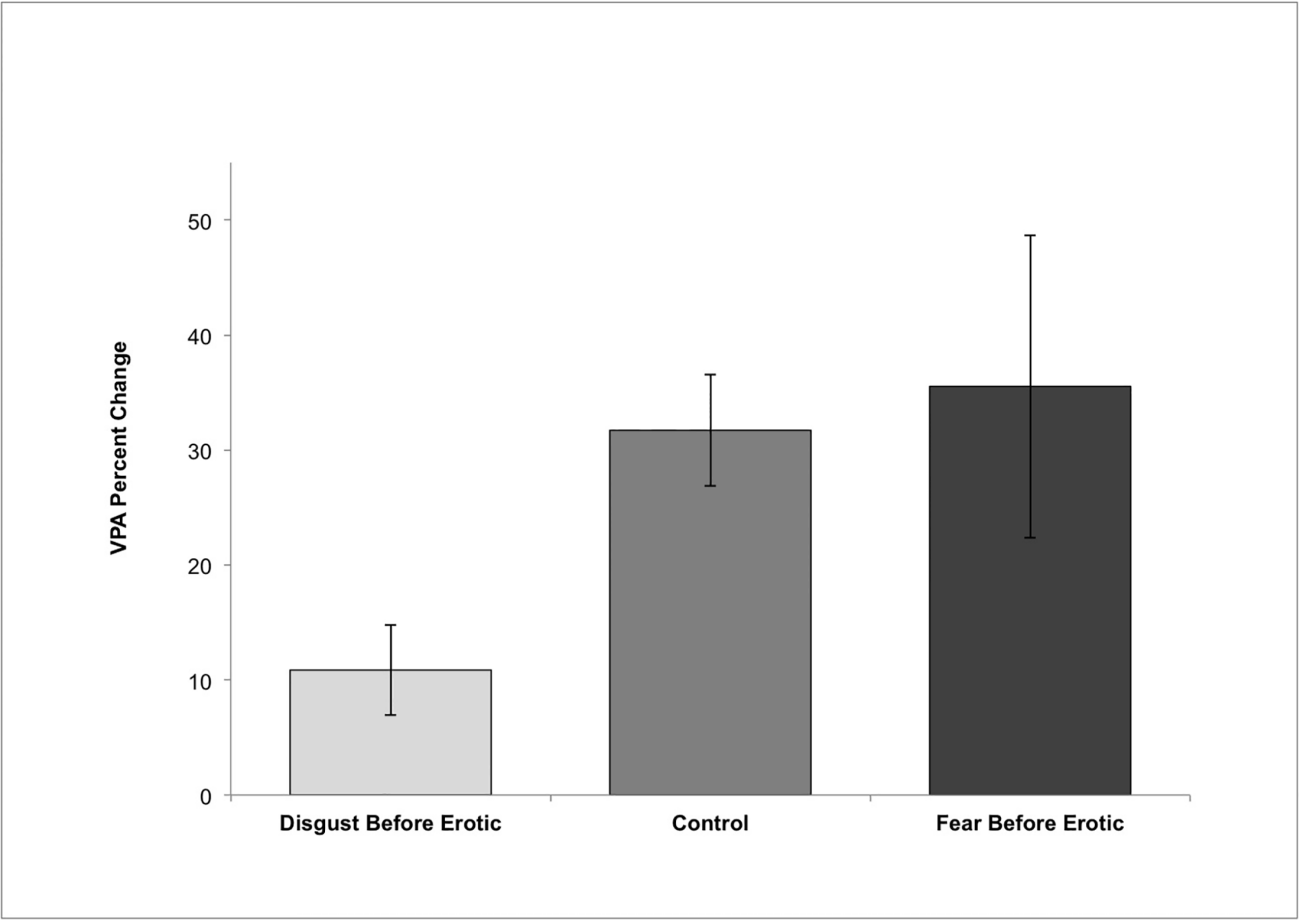
The effects of condition on percent change in VPA. Disgust-before-Erotic, Control (combining two Control-before-Erotic conditions) and Fear-before-Erotic. Error bars are standard error.

To test the effect of emotion induction on sexual arousal, we combined the two conditions in which the erotic film was presented after a neutral film to form a control condition, and then compared this to the conditions in which emotions were induced before the erotic film. The data did not meet homogeneity of variance assumptions; hence a nonparametric test was employed. A Kruskal-Wallis test comparing 76 women in three groups showed a significant difference in percent change across the three groups χ^2^(2) = 5.9, *p*_one tailed_ = .025 (see Fig 2). In planned post-hoc Welch’s t-tests (which do not assume homogeneity of variance) there was a significant difference between the control condition and the Disgust-before-Erotic condition *t*(58.59) = 3.35 *p*_one-tailed_ = .001, *d* = .83 and between the Disgust-before-Erotic and Fear-before-Erotic conditions *t*(16.50) = 1.80, *p*_one tailed_ = .045, *d* = .64 such that women in the Disgust-before-Erotic condition showed the lowest levels of arousal. There was no significant difference between the control and Fear-before-Erotic conditions *t*(17.91) = 0.27, *p* = .79. Using VPA percent change as the dependent variable controlling for disgust propensity, the results were nearly identical χ^2^(2) = 5.6, *p*_one tailed_ = .03, and the pattern of t-test results remained significant. Prediction 1 was supported (Fig 2).

There were no significant differences between conditions on any of the self-report measures (see Table 2) (all ps > .12); controlling for disgust propensity did not change the pattern of results.

**Table 2:** ANOVA values and descriptive statistics for the self-reported sexual arousal measures in each of three conditions n = 63.

	ANOVA F and p values	Disgust before Erotic n = 19	Neutral Before Erotic n = 33	Fear Before Erotic n = 11
Mean Self-Report Sexual Arousal	F = .53 p = 0.59	.43 (.12)	.44(.16)	.49(.15)
Peak Self-Report Sexual arousal	F = .01 p = 1.00	.67 (.18)	.67 (.19)	.66(.19)
Time it took to reach Peak Self-Reported sexual arousal (in seconds from beginning of erotic film)	F = 2.17 p = .12	384 (81)	320 (121)	353 (103)

Prediction 2- If disgust uniquely inhibits sexual arousal, then, for participants in Condition 1 (Disgust-before-Erotic), disgust ratings will negatively correlate with physiological sexual arousal; the same will not be true of other ratings of the same stimuli.

**Fig 3:**
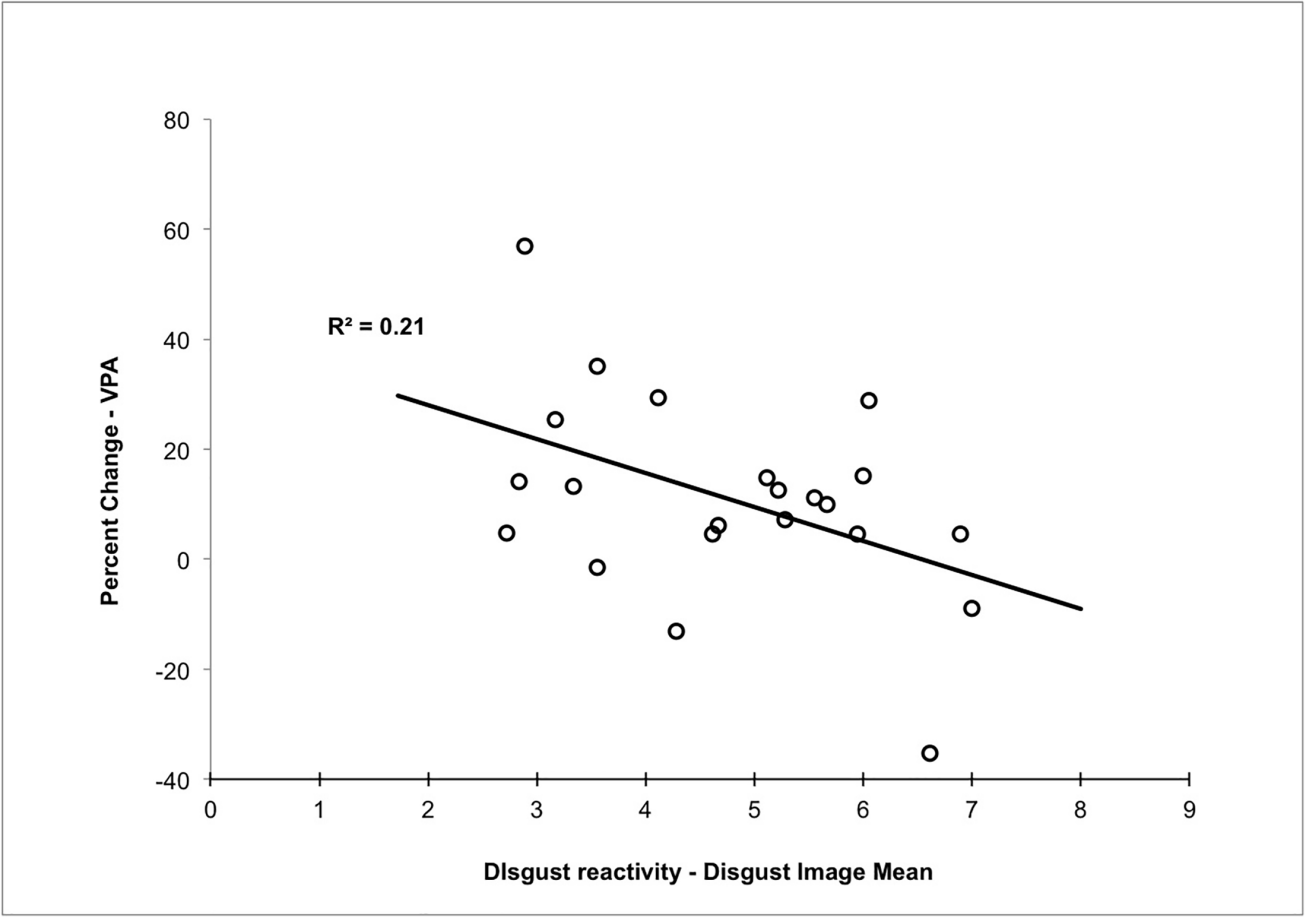
Scatterplot of the correlation for participants in the Disgust-before-Erotic condition between sexual arousal (percentage change in VPA from neutral to erotic stimuli) and the mean of disgust ratings of images. Each point represents one participant.

For analyses involving image ratings, for each participant we first calculated the mean of the image ratings for each emotion. To test Prediction 2 we compiled the Disgust ratings for all participants in the Disgust-before-Erotic condition and correlated this with VPA percent change, finding a significant negative correlation *r*(20) = -.46, *p*_one tailed_ = .02 (see Fig 3); in contrast, no significant correlation occurred between VPA percent change and any of the other ratings: Fear *r*(20) = -.03, *p* = .89; Enjoyment, *r*(19) = .004, *p* = .99; Sexual, *r*(19) = .01, *p* = .95. However, disgust ratings did not correlate significantly with average self-report sexual arousal *r*(17) = -.02, *p* = .95 nor with peak self-report sexual arousal *r*(17) = -.09, *p* = .72. The closest correlation to reaching significance was the relationship between disgust ratings and the amount of *time* it took to reach peak self-reported sexual arousal *r*(17) = .31, *p* = .20.

Prediction 3- If disgust is associated with reduction in sexual function in clinical populations, then disgust propensity may predict lower sexual arousal in Conditions 2 and 3, in which a neutral film is presented before the erotic film.

There was no correlation between disgust propensity and VPA percent change *r*(35) = -.09, *p* = .60 in Conditions 2 and 3. There were also no correlations between disgust propensity and any of the self-reported sexual arousal measures: Average self-reported arousal *r*(29) = -.05, *p* = .79, maximum self-reported arousal *r*(29) = .16, *p* = .38 and time to reach maximum self-reported sexual arousal *r*(29) = -.13, *p* = .49.

#### Predictions 4 and 5 pitted against one another

Prediction 4- If sexual arousal indicates increased risk of disease transmission, then sexual arousal may potentiate disgust reactions. Participants sexually aroused before exposure to disgust stimuli will therefore show higher disgust ratings, and sexual arousal will be positively correlated with disgust ratings.

Prediction 5- If sexual arousal adaptively reduces disgust, then participants who are sexually aroused will show lower disgust ratings, hence participants in the Erotic-before-Disgust condition will show lower disgust ratings than those in other conditions, and their physiological sexual arousal will be negatively correlated with their disgust ratings.

First, we compared Condition 1, in which participants viewed a control film before viewing disgusting images, and Condition 2, in which participants viewed an erotic film before viewing disgusting images. We found no difference in disgust ratings between Condition 1, M = 4.77 SD = 1.36, and Condition 2, M = 5.00 SD = 1.66, *t*(41) = -.46, *p* = .65. Within Condition 2, Erotic-before-Disgust, we found no significant correlation between VPA percent change and disgust ratings *r*(19) = -.16, *p* = .49. We conducted an exploratory analysis of self-reported sexual arousal and disgust reactivity, finding no significant relationship between any of our arousometer ratings (mean self-reported sexual arousal, maximum self-reported sexual arousal, and time to peak self-reported sexual arousal) and subsequent disgust reactivity (all p values > .20). The closest correlation to reaching significance was between maximum self-reported sexual arousal and disgust reactivity *r*(18) = .30 p = .20 such that those women who self-reported greater peak sexual arousal tended to rate subsequent images as more disgusting. Thus we found no evidence that sexual arousal alone increases or decreases disgust reactivity.

**Fig 4:**
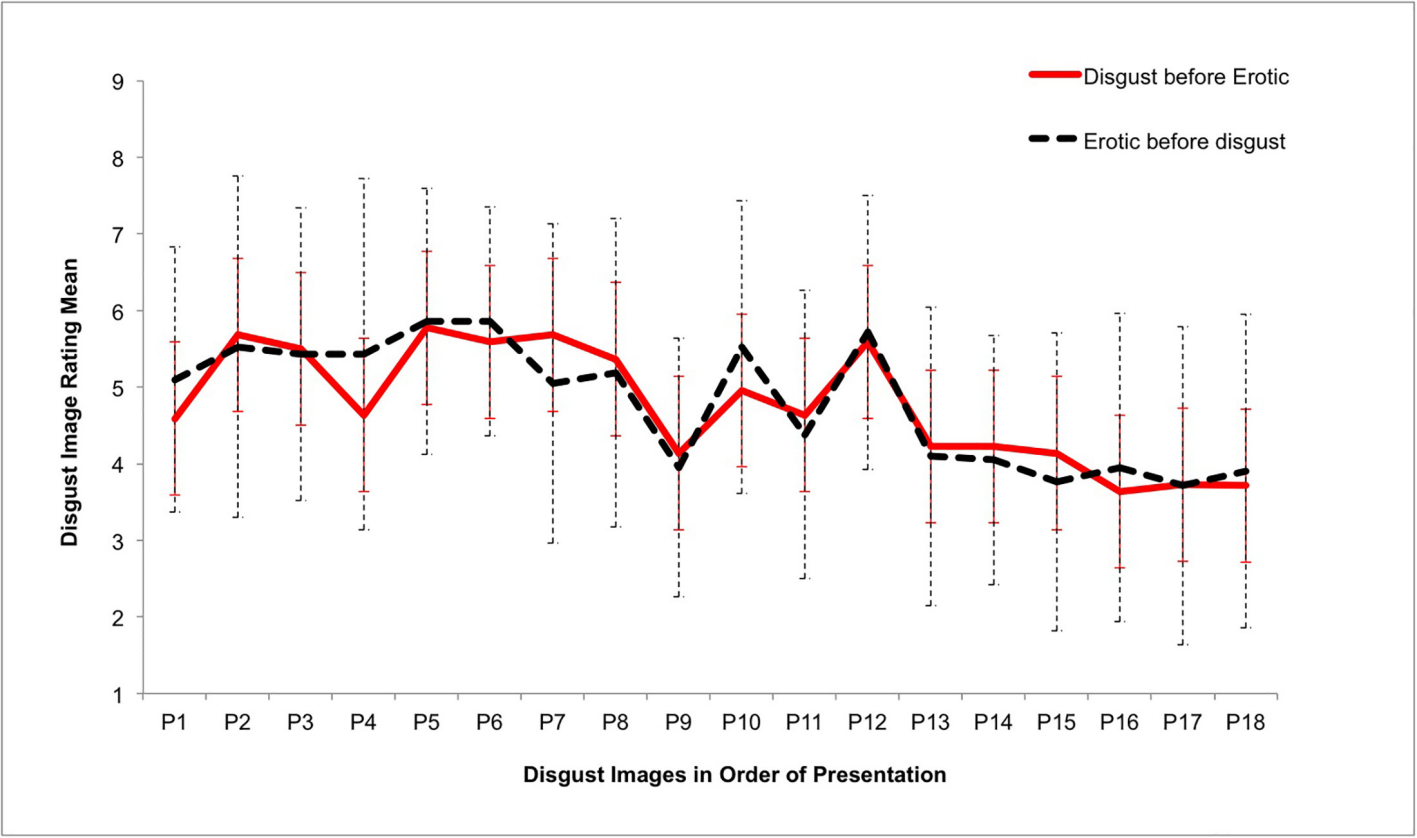
Mean ratings of disgust for disgust images (disgust reactivity) in the Disgust-before-Erotic (n = 22) and the Erotic-before-Disgust (n = 21) conditions showing similar image ratings over time. Error bars indicate +1 or -1 standard deviation.

It is possible that the effect of sexual arousal on disgust sensitivity was short-lived and thus was only present for the first few disgust images presented to participants after the erotic stimuli. In the Disgust-before-Erotic and the Erotic-before-Disgust conditions, disgust images were presented to participants in the same order. Fig 4 shows that there is no discernible difference between the two conditions in disgust reactivity over time, thus indicating that the overall absence of a significant difference in disgust reactivity between the two conditions is not masking a short-lived effect.

**Fig 5:**
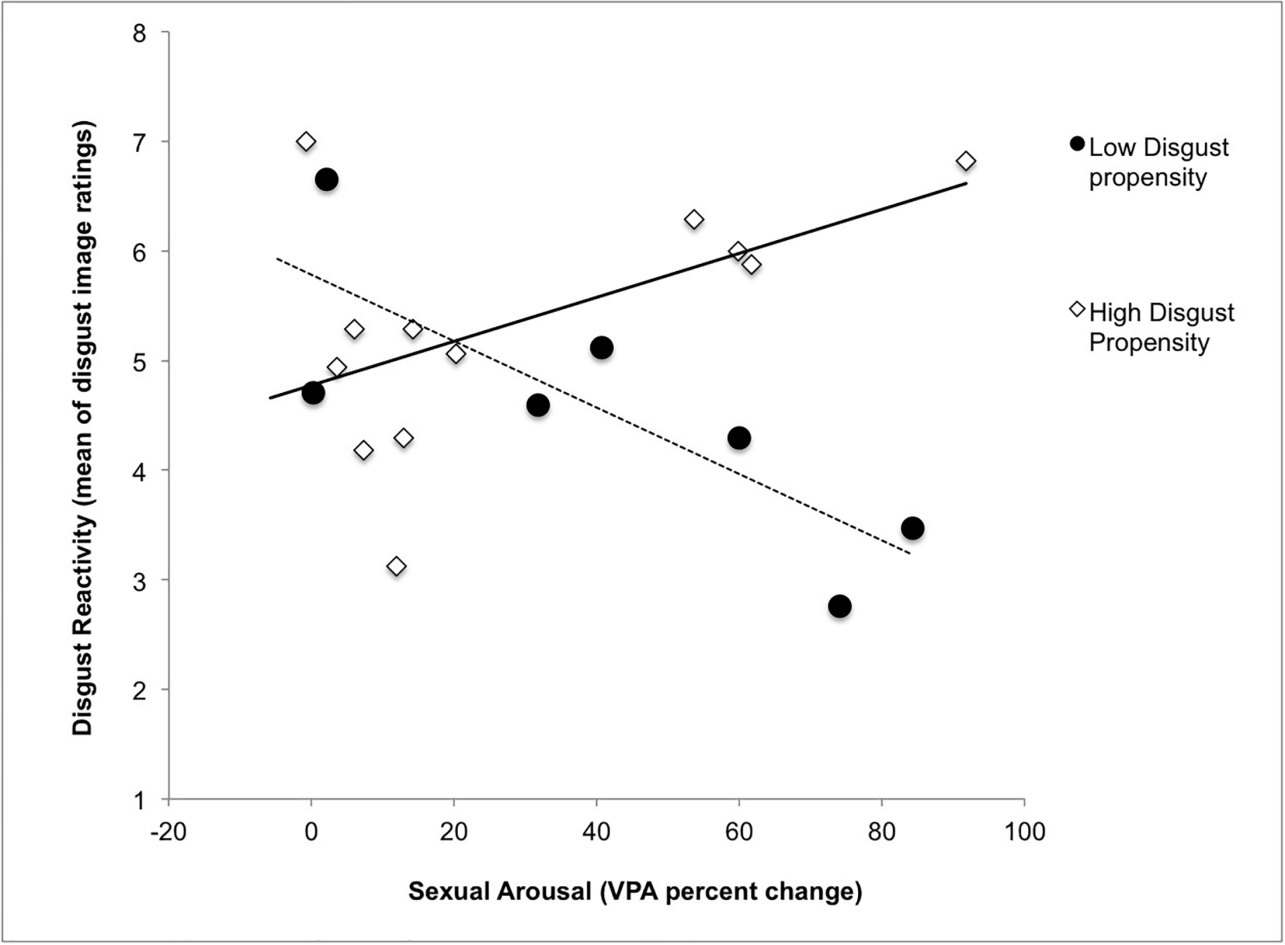
Scatterplot of participants in the Erotic-before-Disgust condition only. The participants are divided by a median split of baseline pathogen disgust propensity showing an interaction trend (β(17) = 1.24, *p* = .08) of baseline pathogen disgust propensity and sexual arousal on ratings of disgust-eliciting stimuli (Y axis).

In light of the well-documented sex difference in disgust propensity noted in the Introduction, we performed two exploratory analyses based on the idea that women low in baseline disgust propensity might respond in a way that is more similar to the male participants in Ariely and Loewenstein’s [21] and Stevenson, Case and Oaten’s [22] previous studies. After centering the variables, we conducted a linear regression and found that the interaction of VPA percent change and baseline disgust propensity nearly significantly predicted disgust image ratings, β(17) = 1.24, *p* = .08, with no main effects of either baseline disgust propensity, β(17) = .047, *p* = .84 or VPA percent change, β (-.16) *p* = .46. We then conducted another exploratory analysis using a median split on disgust propensity. Women high on disgust propensity showed a nearly significant positive correlation between sexual arousal and disgust image ratings *r*(10) = .55, *p* = .07, while women low on disgust propensity displayed a nonsignificant trend in the opposite direction, showing lower disgust ratings as arousal increased *r*(7) = -.53, *p* = .14, see Fig 5.

#### Fear and sexual arousal analyses- exploratory

Fear ratings were higher in the Fear-before-Erotic condition than in the Erotic-before-Fear condition (see Table 1) but this difference did not reach significance *t*(30) = 1.66 *p* = .11, *d* = .61.

In the Fear-before-Erotic condition there was a nonsignificant trend toward a correlation between mean fear rating and VPA percent change, *r*(13) = -.44, *p* = .10. In this condition there were no significant correlations between fear ratings and self-report sexual arousal; this was true of correlations between average self-report sexual arousal and mean fear image ratings *r*(9) = .37, *p* = .26; maximum self-report sexual arousal and fear image ratings *r*(9) = .25, *p* = .46; and time to peak self-report arousal and fear image ratings *r*(9) = .02 p = .95.

In the Erotic-before-Fear condition we found no significant correlation between VPA percent change and fear ratings, *r*(15) = .03, *p* = .92. In this condition there was a trend toward a correlation between how long it took to reach peak self-report sexual arousal and fear ratings *r*(10) = -.56, *p* = .06, such that women who reached peak sexual arousal faster showed a trend in rating the images that followed as more fear-inducing. The pattern of other self-report arousal measures revealed a trend of greater self-reported arousal being associated with a reduction in fear: there was a nonsignificant negative correlation between mean self-report sexual arousal and fear ratings *r*(10) = -.37, *p* = .24, and a similar result appeared with regard to the relationship between maximum self-report sexual arousal and fear ratings *r*(10) = -.40, p = .20.

## Discussion

### Overview of Main Findings

This study was the first to measure the reciprocal effects of disgust elicited by cues of the presence of pathogens and physiological sexual arousal in women. We found that women exposed to disgusting images before erotic stimuli subsequently displayed significantly lower *physiological* sexual arousal, an immediate and unconscious appraisal, in response to erotic stimuli compared to women exposed to a control film or fear-inducing images. Correspondingly, the intensity of disgust experienced, as reported by participants, predicted a *decrease* in physiological sexual arousal in a linear fashion. In contrast, self-report sexual arousal, representing more conscious appraisal of erotic cues, was largely unaffected by previously induced emotions. We did not find that disgust propensity (trait disgust) predicted lower sexual arousal in those conditions in which neutral stimuli were presented before erotic stimuli. We also did not find a straightforward relationship between sexual arousal and subsequent disgust reactivity.

### Sexual Arousal and Disgust Reactivity

Unlike Stevenson et al.’s and Ariely and Lowenstein’s [21] results in men and Borg and de Jong’s [23] results in women, we did not find that sexual arousal had a direct and unmediated effect of reducing subsequent disgust reactivity. Borg and de Jong [23] found that sexually aroused women completed more disgusting tasks and rated these tasks as less disgusting but Lee et al. [24] found that sexually aroused women were more disgusted by cues of pathogen disgust. However, we found no difference between participants who viewed a neutral film and those who viewed an erotic film in ratings of subsequent disgusting stimuli (disgust reactivity), and this was true even in regard to ratings of those images shown immediately after the erotic film. We also did not find a significant negative relationship between physiological sexual arousal and disgust reactivity. In an exploratory analysis we found a non-significant positive correlation between self-reported peak sexual arousal (i.e. maximum self-reported arousal) and disgust reactivity lending partial support to the idea that sexual arousal can increase core disgust. Thus, the evidence that sexual arousal decreases disgust reactivity in women is mixed. It is unclear which of the many differences between our study and that of Borg and de Jong [23] accounted for the difference in findings. For example, Borg and de Jong’s Dutch participants iteratively viewed pornography for a longer period and interacted with disgust stimuli directly. In contrast, North American participants in our study viewed pornography for a shorter period of time and rated photographic disgust stimuli while undergoing physiological measurement of sexual arousal. Lee et al. [24] found the American women in their online study (who could choose their own preferred method for achieving sexual arousal in their homes) showed increased disgust at descriptions of pathogen cues. Our results show some indication that baseline trait disgust could predict whether sexual arousal increases or decreases disgust reactivity.

### Disgust Propensity’s Influence on Disgust Reactivity

Individual differences in disgust propensity may reflect differences in vulnerability to infection (e.g., [46]). Thus, low disgust sensitivity may index a favorable cost-to-benefit ratio for sexual behavior for the given individual, such that the benefits of reproduction outweigh the costs of potential infection. For instance, Murray et al. [32] found an intensified correlation in women between perceived vulnerability to disease (which overlaps with trait disgust) and lower desire to engage in promiscuous sex in a disease salience condition. Previously, Borg and de Jong [23]found that, although trait disgust (measured through the Disgust Propensity and Sensitivity Scale Revised [53]) predicted both self-reported disgust reactions and willingness to engage in tasks designed to elicit disgust, nevertheless, there was no significant interaction between trait disgust and experimental induction of sexual arousal in predicting either of these indices of disgust reactivity.

Examining the effects of physiological sexual arousal on reactions to disgust elicitors, we found hints that the trait-level propensity to experience disgust in response to cues of the presence of pathogens may interact with physiological sexual arousal, enhancing disgust reactions in highly disgust-prone individuals. Although the effect of disgust *propensity* on the degree to which sexual arousal influences disgust *reactivity* did not reach statistical significance, there is reason to believe that this may have been due to features of our methods, as 1) we did not design our sample so as to capture a representative distribution of disgust propensities, and 2) our sample size for the comparisons in Condition 2 is small. In light of these limitations, and in light of the theoretical considerations mentioned above, we encourage future investigators to more fully explore the role that trait-level disgust propensity plays in the interactions between sexual arousal and disgust response.

Among participants who did not undergo emotion induction prior to exposure to erotic stimuli, we found no evidence that disgust propensity by itself inhibits sexual arousal howsoever measured. Grauvogl et al. [16] also did not find that any of three different measures of trait disgust predicted genital sexual arousal to pornography in a sample of women with normal sexual functioning. In both cases this may be due to selection bias for women with good sexual functioning, volunteer bias due to the nature of the study protocol, and no selection criteria to capture a breadth of disgust propensities.

### Examination of Fear Conditions

Overall, women in the Fear-before-Erotic condition showed higher VPA than women in the Disgust-before-Erotic condition. However, within the Fear-before-Erotic condition, fear ratings were non-significantly related to decreased physiological sexual arousal. Thus, there is some evidence that our more naturalistic fear stimuli did have a negative impact on sexual arousal, albeit less so than the influence of disgust. There were no significant correlations between fear ratings and self-reported sexual arousal in the Fear-Before-Erotic condition.

Women in the Erotic-before-Fear condition showed lower fear responses than women in the Fear-before-Erotic condition, although VPA did not correlate significantly with reduced fear responses in the former. Both maximum self-reported sexual arousal and mean self-reported sexual arousal showed nonsignificant negative correlations with fear ratings. One possibility that should be explored in future research is that sexual arousal decreases the fear response to natural threats such as those depicted in our stimuli. However, we also found a nearly significant trend in the opposite direction, those women who self-reported maximum sexual arousal faster tended to rate subsequent fear images as *more* frightening.

### Applications, Limitations and Future Directions

Our findings confirmed our prediction that disgust, an emotional state that impels individuals to avoid contexts associated with a risk of disease transmission, would reduce sexual arousal. This could have implications for the sexual functioning of those who are easily disgusted, or who are more often exposed to disgust-eliciting stimuli in day-to-day life. While there is some evidence that those who work closely with a given disgust stimulus can become desensitized [54], the current study points to the admittedly speculative possibility that chronic exposure to disgusting stimuli could reduce the capacity to become sexually aroused. Disgust has been implicated in asexuality [55], sexual aversion [56], hypoactive sexual desire [57], as well as vaginismus and dyspareunia [25,33]. As our study is the first to point to the specific effect of pathogen disgust elicitation on subsequent sexual arousal, to determine the extent of the clinical consequences of this pattern, in the future it will be important to investigate how long this effect lasts.

One weakness of the current study is our small sample size. Because of the difficulty of recruitment for psychophysiological studies of sexual arousal and the time-intensive nature of this kind of research, most prior investigations addressing such topics have employed samples of around 15–25 per group (e.g., [16,58–60]). We predicted that our core predictions should yield large effect sizes hence power estimation suggested that these predictions could be tested using samples sizes typical of psychophysiological studies of sexual arousal. In contrast, many of our within-condition predictions and results must be considered preliminary until larger samples can be employed.

A principal strength of the current study compared to previous work examining the relationship between sexual arousal and disgust is our use of a direct physiological measure of sexual arousal. That said, physiological measures are not without their limitations, as, for example, their invasive nature may generate self-selection among participants [61,62]. Accordingly, a combination of different types of measures may prove optimal. Relatedly, our use of physiological measures to assess sexual arousal contrasts with our reliance on self-reports in assessing other emotional states in this study. Although the physiological assessment of emotions such as fear and disgust is still developing, work using facial electromyography (e.g.,[63]; see, for example [25]), or pupil dilation [64] appears promising. Future investigations may therefore improve on the present work by employing physiological measures of both sexual arousal and emotional state.

Some previous research has controlled for the potentially positive valence of the erotic videos [22,23] or for physiological arousal [24]. In their study of women, Borg and de Jong [23] found that, compared to participants in the neutral stimulus condition, those in the sexual arousal condition did show lower disgust reactions to nonsexual disgust elicitors; however, although there was a strong trend, there was no significant difference between participants exposed to erotic stimuli and those in a positive valence control group, suggesting that the positive valence of sexual arousal may be an important factor in decreasing disgust reactivity. In contrast, however, Stevenson et al. [22] also employed a positive valence control condition in their study of men, yet found no effect of condition on reactions to nonsexual disgust elicitors. Clearly, future research exploring the relationship between sexual arousal and disgust reactivity should employ both a measure of baseline individual differences in disgust propensity and a positive valence control condition.
